# Astrovirus in the Brazilian Amazon: First detection of non-classical astroviruses (MLB-3) in the Americas

**DOI:** 10.1371/journal.pone.0352094

**Published:** 2026-07-01

**Authors:** Dan Santos Alves, Dielle Monteiro Teixeira, Edivaldo Costa Sousa Junior, Danielle Rodrigues de Deus, Patrícia dos Santos Lobo, Sylvia de Fátima dos Santos Guerra, Luciana Damascena da Silva, Hugo Reis Resque, Luana Silva Soares, Yvone Benchimol Gabbay, Jones Anderson Monteiro Siqueira

**Affiliations:** 1 Laboratório de Vírus Gastroentéricos, Seção de Virologia, Instituto Evandro Chagas, Ananindeua, Pará, Brasil; 2 Laboratório de Bioinformática, Seção de Parasitologia, Instituto Evandro Chagas, Ananindeua, Pará, Brasil; 3 Centro de Ciências Biológicas e da Saúde, Universidade do Estado do Pará, Belém, Pará, Brasil; Institut Pasteur de Madagascar, MADAGASCAR

## Abstract

Human astrovirus (HAstV) is a viral agent responsible for acute gastroenteritis (AGE), primarily affecting children and the elderly worldwide. Belonging to the *Astroviridae* family, HAstV is classified into eight classical serotypes (HAstV 1–8) and two other divergent non-classical clades: Melbourne (MLB 1–3) and Virginia (VA 1–6), which have been associated with gastroenteritis, central nervous system complications, and acute respiratory disease. This study aimed to investigate the frequency of classical and non-classical HAstV in fecal specimens collected from children up to 14 years of age in northern Brazil, within the Amazon region, between 2013 and 2022. A total of 560 samples, all previously tested negative for other gastroenteric viruses such as rotavirus and norovirus, were analyzed using reverse transcription followed by quantitative polymerase chain reaction (RT-qPCR) and conventional RT-PCR. For classical HAstV, 10.7% (60/560) of the samples were positive by RT-qPCR and 2.0% (11/560) to 3.0% (17/560) by conventional RT-PCR using different primers set. Non-classical HAstV was detected in 0.2% (1/560) of the samples. Diarrhea was present in 91.7% of positive cases, vomiting in 71.7%, and fever in 53.3%. The most affected age group was children aged >5–10 years (25.0%), with no significant association between infection rate and sex. A higher number of infections occurred during the Amazon winter (11.6%), with Roraima identified as the federative unit with the highest number of cases. Fifteen samples (88.2%, 15/17) were sequenced and identified as classical HAstV, with the following genotypes detected: HAstV-1 (60.0%, 9/15), HAstV-3 (20.0%, 3/15), and HAstV-4 (20.0%, 3/15). Non-classical HAstV sequencing was performed on 12 (93.2%, 12/13) positive specimens, characterized as HAstV-1 (50%, 6/12), HAstV-3 (25%, 3/12), HAstV-4 (16.7%, 2/12), and HAstV-MLB-3 (8.3%, 1/12). A probable recombinant strain was identified, classified as HAstV-4 based on the ORF2 region and HAstV-1 based on the ORF1b region. This study provides updated epidemiological data on HAstV in the Brazilian Amazon and highlights the genetic diversity of both classical and non-classical genotypes. Notably, it reports the first detection – and the second complete genome repository – of the rare MLB-3 genotype in the Americas.

## Introduction

Acute non-bacterial gastroenteritis, also known as viral gastroenteritis, refers to diseases of the intestinal tract caused by various enteric viruses. These illnesses can occur in both epidemic and sporadic settings, affecting individuals across all age groups and contributing to significant morbidity and mortality [1,2]. The primary signs and symptoms reported in individuals infected with these viruses include nausea, vomiting, diarrhea, fever, general malaise, abdominal pain, and headache. In most cases, the clinical presentation is mild [3,4].

Rotaviruses and noroviruses are the primary viral agents responsible for acute gastroenteritis (AGE) and account for numerous hospitalizations worldwide. However, other enteric viruses have also been identified as important contributors to this disease [5,6]. Human astroviruses (HAstV) are among these agents and are known to cause AGE across all age groups, particularly affecting children under two years of age, with more severe outcomes observed in the elderly and immunocompromised individuals [7].

HAstV belong to the *Astroviridae* family, genus *Mamastrovirus*, and are composed of eight distinct serotypes, commonly referred to as classical types. However, since 2008, new species have been reported and grouped into two distinct clades, named Melbourne (MLB) and Virginia/Human-Mink-Ovine-like (VA/HMO), which are classified as non-classical astroviruses (AstV) [8]. AstV is a non-enveloped, spherical virus with a genome consisting of a single-stranded, positive-sense ribonucleic acid (^+^_ss_RNA) of approximately 6.8 kilobases (kb). This genome is divided into three open reading frames (ORFs): ORF1a, ORF1b, and ORF2 [9].

Transmission occurs primarily via the fecal-oral route, through the consumption of contaminated food and water, or by direct person-to-person contact. It is commonly associated with outbreaks in semi-enclosed environments with large crowds [10]. The main symptoms of HAstV infection include vomiting, diarrhea, abdominal pain, and fever. However, complications involving the central nervous system and acute respiratory disease have also been reported, particularly in association with non-classical AstV strains [11].

HAstV has a cosmopolitan distribution and is considered a common cause of childhood AGE in developing countries, with an average prevalence ranging from 3% to 5% [7,12]. In Brazil, circulation patterns are similar to those observed in other countries, with an average positivity rate of 7%, which may exceed 50% during outbreaks [13,14]. Studies have demonstrated the circulation of HAstV in pediatric populations with acute gastroenteritis, although generally at low frequencies, accompanied by notable genetic diversity.

In the Amazon region, a retrospective study conducted in Rio Branco, Acre, reported an overall HAstV detection rate of 4.7%, with infections identified across all age groups and a predominance in children under two years of age; molecular characterization revealed the circulation of genotypes HAstV-1a and HAstV-2c [15]. Similarly, in Belém, Pará, a three-year surveillance study in hospitalized children identified a positivity rate of 3.9%, with a predominance of HAstV-1a, followed by HAstV-2 (including subtypes 2b and 2c), as well as the detection of genotypes HAstV-3c and HAstV-4c, which had not been previously reported in Brazil [16]. Collectively, these findings highlight the co-circulation of multiple classical HAstV genotypes in the Brazilian Amazon, reinforcing the importance of continued molecular surveillance to better understand the epidemiology and genetic diversity of this virus in the country.

To date, cases of severe gastroenteritis are managed primarily with intensive oral or intravenous rehydration due to the absence of specific antiviral treatments – a situation further compounded by the lack of commercially available vaccines. Despite their clinical significance, astroviruses remain among the least studied RNA enteric viruses [17–19].

In this context, maintaining epidemiological surveillance of HAstV is crucial, as monitoring its prevalence and identifying emerging viral strains in the Amazonian pediatric population may contribute to a better understanding of infection patterns and associated epidemiological profiles. These infections can result in hospitalizations and generate costs for both public and private healthcare systems, with further social and economic impacts on affected families due to ongoing transmission chains [20,21].

This study aimed to investigate the occurrence and genetic diversity of classical and non-classical HAstV in fecal specimens collected from children served by the Viral Gastroenteritis Surveillance Network in the Northern region of Brazil over a 10-year period (2013–2022).

## Materials and methods

### Study area

The samples used in this study were collected between 2013 and 2022 from the Northern region of Brazil, encompassing the federative units of Acre, Amazonas, Roraima, Rondônia, Pará, Amapá, and Tocantins, which together account for approximately 77.8% of the Brazilian Amazon.

This region is characterized by a warmer and more humid climate compared to other parts of the country, with high temperatures throughout the year, particularly during the local summer (June to November) [22]. The estimated population is 17.4 million inhabitants, of whom only 2.5 million (14.4%) have access to a sewage system and 10.8 million (62.1%) to a piped water supply [23].

### Sample selection

This study is a retrospective meta-analysis surveillance involving fecal samples tested for HAstV detection, conducted in accordance with ethical principles for research involving human subjects and approved by the Research Ethics Committee (registration number 1,856,561). A total of 2,003 fecal samples from pediatric patients of both sexes were received through the Rotavirus Diarrheal Disease Epidemiological Surveillance Network. This network is part of the Acute Diarrheal Disease Monitoring system, a sentinel surveillance program that has been active in all Brazilian states since 1994. It involves three institutions: Fundação Oswaldo Cruz (Fiocruz), Instituto Adolfo Lutz (IAL), and Instituto Evandro Chagas (IEC).

Of the 2,003 samples, 560 (28%) were selected for this study based on the following inclusion criteria: a negative diagnosis for other gastroenteric viral agents, such as rotavirus and norovirus, and patient age ranging from 0 to 14 years. Samples were excluded if they had insufficient volume for laboratory procedures or contained inaccurate or incomplete identification data.

Information regarding the location and date of sample collection, as well as epidemiological data such as age, sex, and possible symptoms, was extracted from a database populated by epidemiological records submitted by the Central Public Health Laboratories of the Brazilian federative units responsible for sending the samples to the IEC. Patient data were accessed throughout 2023, with individual information that could identify the participants included in this research being protected.

### Sample processing

Fecal suspension samples were prepared in an elution buffer (Tris/HCl/Ca² ⁺ 0.01 M, pH 7.2) at a concentration of 10% weight/volume. Total nucleic acid extraction was performed using the silica column method with the commercial QIAamp Viral RNA Mini Kit (QIAGEN^®^), following the manufacturer’s instructions. All samples were stored at temperatures ranging from −20 °C (temporary storage) to −70 °C (permanent storage) for later use.

### Virus detection

To detect classical HAstV viral RNA, the samples were tested using quantitative reverse transcription polymerase chain reaction (RT-qPCR) with the GoTaq Probe 1-Step RT-qPCR System kit (Promega^®^). The assay utilized the forward primer AstV-F (5’-CCDGCCAGRCTCACAGAAGAG-3’), reverse primer AstV-R (5’-GACTTGCTAGCCATCACACTYC-3’), and a probe (5’-HEX-ACTCCATCGCATTTGGAGGGAGGACCBKRQ-3’) (76 bp), as described by Dai et al. [24]. These primers and probe target a highly conserved region in the overlapping zone of ORF1b and ORF2.

Detection of non-classical HAstV by RT-qPCR was not possible due to the unavailability of reagents for detecting the MLB and HMO/VA clades using this method in the laboratory where the study was conducted. For the conventional detection of non-classical HAstV, all 560 fecal samples were analyzed using the forward primer SF0073 (5’-GATTGGACTCGATTTGATGG-3’) and reverse primer SF0076 (5’-CTGGCTTAACCCACATTCC-3’) (409 bp), as described by Finkbeiner et al. [25]. This assay also enabled the detection and genotyping of classical HAstV.

### Genotyping

HAstV was detected by conventional RT-PCR using two different sets of primers to identify classical and non-classical HAstV. The commercial One-Step AccessQuick RT-PCR System (Promega^®^) was used, following the manufacturer’s guidelines, with specific reagents and cycling parameters for each viral group analyzed.

For the genotyping of classical HAstV, we used the forward primer Mon269 (5’-CAACTCAGGAAACAGGGTGT-3’) and the reverse primer Mon270 (5’-TCAGATGCATTGTCATTGGT-3’) (449 bp), which target a conserved region in the 5’ portion of ORF2. This assay is capable of detecting all eight classical HAstV serotypes known to date [26]. The genotyping of classical and non-classical HAstV was carried out using the same primer set applied in the detection step (SF0073 and SF0076).

#### Partial sequencing and phylogenetic analysis.

Samples identified as positive by RT-PCR for classical and non-classical HAstV were purified using the MEGAquick-spin™ Total Fragment DNA Purification Kit (Intron^®^ Biotechnology), following the manufacturer’s instructions. The purified products were then subjected to Sanger sequencing using the ABI Prism 3130xl DNA Genetic Analyzer System (Applied Biosystems, Foster City, USA).

The partial sequences obtained were edited using BioEdit v7.2.5 and compared with similar sequences available in the GenBank database (National Center for Biotechnology Information, USA) using the BLAST tool, with a preference for complete nucleotide sequences as the selection criterion. Sequences were aligned using Geneious v10.0.8 [27], and phylogenetic trees were constructed using the maximum likelihood method and the Tamura 3-parameter substitution model [28], incorporating a gamma distribution (+G) and a proportion of evolutionarily invariant sites (+I). The best-fit model was determined using the model selection tool in MEGA v11.0.13, and tree reliability was assessed through a non-parametric bootstrap analysis with up to 1,000 replicates. Sequences from the *Avastrovirus* genus (MW826482/NC_003790.1) were used as the outgroup.

### Complete genome sequencing

One sample suggestive of a non-classical HAstV genotype was submitted for whole-genome sequencing using a target enrichment approach based on hybrid capture. Library preparation was performed from total RNA, incorporating both pre-enrichment and enrichment steps using the Illumina DNA Prep with Enrichment kit (Illumina, San Diego, CA, USA), the IDT for Illumina^®^ DNA/RNA UD Set C (Illumina, San Diego, CA, USA), and Viral Surveillance Panel probes (Illumina, San Diego, CA, USA), all according to the manufacturer’s instructions.

Quality control was conducted using the High Sensitivity D1000 ScreenTape Assay on the Agilent 2200 TapeStation system (Agilent Technologies, Waldbronn, Germany). The library was sequenced on the Illumina MiSeq™ platform with a read length of 2 × 150 bp.

Raw reads were trimmed to remove low-quality bases using the FASTP preprocessor [29]. Sequence alignment was performed with MAFFT v7 (https://mafft.cbrc.jp/ alignment/software/), and genome editing was done using Geneious v10.0.8 [27]. Genome assembly was carried out using a *de novo* strategy with MEGAHIT v1.2.9 (https://software.cqls.oregonstate.edu/updates/megahit-1.2.9/). Taxonomic annotation was performed using Kraken 2 with the Standard database (16 GB) [30].

### Statistical analysis

Statistical analysis was performed using BioEstat version 5.0 [31]. The G-test was applied to assess the significance of signs and symptoms presented by individuals affected by the virus, as well as associations with age range and the spatial and temporal distribution of the cases investigated. The Chi-square test (χ²) was used to evaluate the seasonality of HAstV infections and the sex of the patients. The Kolmogorov-Smirnov test was employed to assess the normality of the age data. If the data were normally distributed, the arithmetic mean was used as the descriptive measure; otherwise, the median was adopted. For all hypothesis tests, a 95% confidence interval (CI) and a significance level of ≤ 0.05 were used.

## Results

### Epidemiological profile

A total of 560 samples collected between 2013 and 2022 were analyzed, and HAstV was detected in 10.7% (60/560) of the cases by RT-qPCR. Regarding the frequency of fever, vomiting, and diarrhea, the symptoms were observed in 53.3% (G = 2.1598; *P* = 0.1417), 71.7% (G = 4.8143; *P* = 0.0282), and 91.7% (G = 7.2399; *P* = 0.0071) of the affected children, respectively (Table 1).

**Table 1:** Detection of Astrovirus by Quantitative Reverse Transcription Polymerase Chain Reaction (RT-qPCR) According to Clinical Signs, Sex, and Age Group in Children with Acute Gastroenteritis in Northern Brazil, 2013–2022.

Characteristics			Positivity (%)
	Symptoms		
	HAstV - Positive	HAstV - Negative	
Fever	32/60	212/500	53.3/42.4
Vomiting	43/60	281/500	71.7/56.2
Diarrhea	55/60	455/500	91.7/91
**Sex**			
Female	31/260		11.9
Male	29/300		9.7
**Age (Years)**			
≤ 5	49/491		10.0
>5 - 10	6/24		25.0
>10 - 14	0/13		0.0
Not informed	5/32		15.6
**Total**	**60/560**		**10.7**

With respect to sex, 260 (46.4%) samples were from female children and 300 (53.6%) from male children, with detection rates of 11.9% (31/260) and 9.7% (29/300), respectively (*χ²* = 0.524; *P* = 0.4691). Among HAstV-positive individuals, the median age was 1 year and 7 months (non-parametric data, Kolmogorov-Smirnov, *P* < 0.01). The most affected age group was > 5–10 years, accounting for 25.0% (6/24) of cases (G = 3.3013; *P* = 0.0690), followed by the ≤ 5 years age group, with 10.0% (49/491) of cases (G = 0.7627; *P* = 0.3825) (Table 1).

### Spatial and temporal distribution of cases

During the study period, the highest frequency of cases was observed in the Brazilian state of Roraima (35.7%, 5/14) (*G* = 4.8482; *P* = 0.0277), while the lowest was in Tocantins (7.7%, 8/104) (*G* = 0.9154; *P* = 0.3387), with no positive cases recorded in Rondônia. The highest positivity rate occurred in 2021 (23.1%, 6/26) (*G* = 2.5309; *P* = 0.1116), while the lowest was in 2022 (3.7%, 1/27) (*G* = 0.9321; *P* = 0.3343). No positive cases were detected in 2020 (Table 2).

**Table 2:** Spatial and Temporal Distribution of Astrovirus Cases Detected by Quantitative Reverse Transcription Polymerase Chain Reaction (RT-qPCR) in Children with Acute Gastroenteritis in the North Region of Brazil, 2013–2022.

Federation Unit	Positive/Total	%
Acre (AC)	3/27	11.1
Amazonas (AM)	31/305	10.2
Amapá (AP)	4/45	8.9
Pará (PA)	9/59	15.3
Rondônia (RO)	0/6	0.0
Roraima (RR)	5/14	35.7
Tocantins (TO)	8/104	7.7
**Total**	**60/560**	**10.7**
**Study period**	**Positive/Total**	**%**
2013	4/62	6.5
2014	4/83	4.8
2015	3/60	5.0
2016	14/82	17.1
2017	15/83	18.1
2018	6/62	9.7
2019	7/66	10.6
2020	0/9	0.0
2021	6/26	23.1
2022	1/27	3.7
**Total**	**60/560**	**10.7**

**Fig 1:**
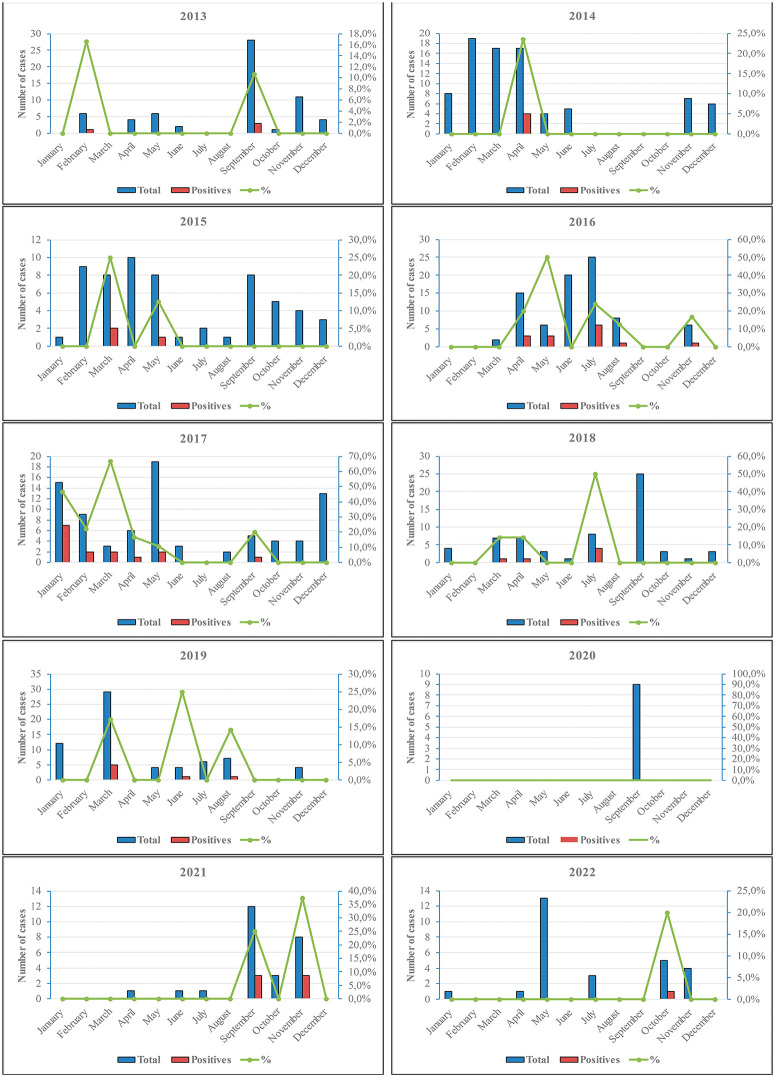
Monthly distribution of positive astrovirus cases in 560 fecal samples from children with gastroenteritis in Northern Brazil, from 2013 to 2022.

The monthly distribution showed positivity peaks in March 2017, with 66.7% (2/3) of cases, followed by July 2018 with 50% (4/8), and May 2016 with 50% (3/6). Regarding seasonality, no consistent patterns of circulation were observed across the months during the ten-year investigation period. However, when data were grouped by season, HAstV was more frequently detected from December to May ‒ a period known as the Amazonian winter due to increased rainfall ‒ with a detection rate of 11.6% (35/303), compared to 9.7% (25/257) during the Amazonian summer (June to November) (*χ²* = 0.3120; *P* = 0.5767) (Fig 1).

### Genotyping

Conventional PCR using the Mon269/Mon270 primer set for canonical HAstV genotyping yielded a positivity rate of 3.0% (17/560), equivalent to 28.3% (17/60) relative to the cases detected by RT-qPCR. Of these, 15 samples (88.2%, 15/17) generated high-quality sequences. The most prevalent genotypes were HAstV-1 (60.0%, 9/15), followed by HAstV-3 and HAstV-4 (each with 20.0%, 3/15) (S1 Fig).

**Fig 2:**
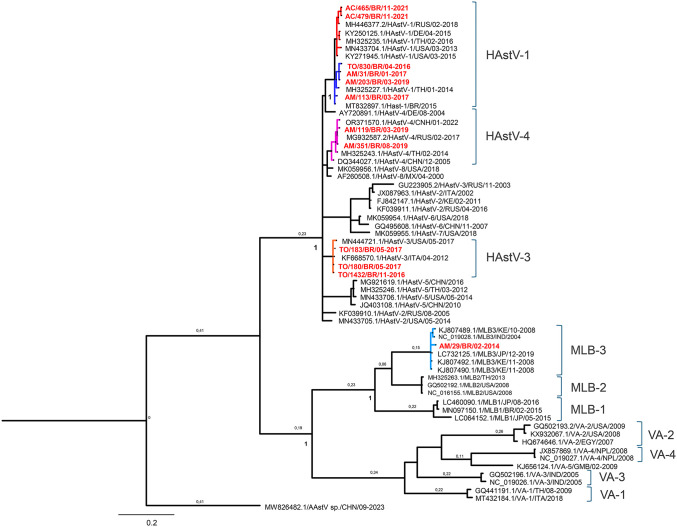
Phylogenetic tree reconstructed from 63 partial ORF1b sequences (305 bp) of classical and non-classical HAstV strains detected in fecal samples from children with gastroenteritis in Northern Brazil, from 2013 to 2022. The tree was constructed using the Maximum Likelihood method with nonparametric bootstrap testing (1,000 replicates), and the evolutionary history was inferred by Bayesian inference (MrBayes) using the GTR substitution model in Geneious software. Markov Chain Monte Carlo (MCMC) chains were run for over 1,000,000 iterations with a 10% burn-in. Convergence was achieved with an ESS value of 504 and an autocorrelation time (ACT) of 1,784.5. Branch lengths are scaled to the number of nucleotide substitutions per site. Strains analyzed in this study are highlighted in red and identified by Brazilian federative unit, sample number, country, and collection date.

Using conventional RT-PCR with SF0073/SF0076 primers ‒ capable of detecting both classical and non-classical HAstV ‒ a positivity rate of 2.3% (13/560) was observed, corresponding to a recovery rate of 20% (12/60) when compared to RT-qPCR. Among these, classical HAstV accounted for 2.0% (11/560), and non-classical HAstV for 0.2% (1/560). Twelve samples (92.3%, 12/13) produced high-quality sequences, and one sample was not sequenced. The predominant genotypes were: HAstV-1 (50%, 6/12), HAstV-3 (25%, 3/12), HAstV-4 (16.7%, 2/12), and MLB-3 (8.3%, 1/12) (Fig 2).

One sample (AM/113/BR/03–2017) was found to exhibit dual characterization following sequencing using two primer sets. It was identified as HAstV-4 based on the ORF2 region, with 97.59% similarity to strains reported in Italy from 2002 and 2002–2005 in children under five with AGE (KC915035.1 and GU216586.1) [32,33], and in Brazil in 2004 (DQ381505.1) [34]. The same sample was also classified as HAstV-1 based on the ORF1b region, showing genetic similarity of 97.33% to a strain identified in Italy in 2002 (KC915035.1) [32], 96.52% to a strain from Italy in 2000 (KY744137.1), and 96.01% to a strain from Nepal, detected between 2010 and 2015 in fecal samples from children under three years old (MW341104.1) [35].

### HAstV-MLB-3 complete genome

The sample, classified as likely belonging to the non-classical HAstV-MLB-3 genotype, was subjected to whole-genome sequencing using target enrichment via a hybrid capture method, which confirmed this classification (S2 Fig). A total of 306,131 reads were obtained prior to trimming, and 305,287 reads remained after trimming. A total of 514 contigs were generated, ranging in length from 232 to 99,844 base pairs, with an average coverage of 1103.4 × . The GC content was 29.9%, and the final genome length was 6,117 base pairs. The nucleotide identity of this sample was 97.6% when compared to the prototype strain NC_019028.

### Access numbers

The sequences obtained in this study have been submitted to GenBank (https://www.ncbi.nlm.nih.gov) under the following accession numbers: partial genes –PQ476025, PQ496858 to PQ496878, and PQ541068 to PQ541070; complete genome –PV088328.

## Discussion

This study investigated the frequency and genetic diversity of HAstV in fecal samples from children with acute gastroenteritis (AGE) living in the Northern region of Brazil between 2013 and 2022. The RT-qPCR positivity rate observed in the present study was 10.7%, which was higher than that reported in a 12-month study conducted in Spain, where 718 fecal samples from children under 10 years of age were analyzed and an overall positivity rate of 5.5% was found [36]. Similarly, a study conducted in Kenya between 1999 and 2005 also reported a lower prevalence of HAstV infection, based on the analysis of 476 children under 10 years of age, with an especially low frequency among those aged 5–10 years (0.2%) [37].

In Brazil, since the first report of HAstV in 1985 [38], several studies have been conducted using fecal samples from children under five years of age in the Northern region, employing conventional molecular biology techniques. Over the years, these studies have demonstrated the presence of this virus, with reported detection rates including: 5.4% between 1990 and 1992 [39]; 3.9% between 2008 and 2011 [16]; 4.7% in 2012 [15]; and 2.4% in 2020 [40].

The variation in HAstV detection rates may be attributed to several factors, including the epidemiological characteristics of the studied regions, the diagnostic methods used, the number of susceptible individuals, socioeconomic conditions, and cases of co-infection with other gastroenteric viruses [41,42].

Regarding the signs and symptoms associated with HAstV-positive cases, diarrhea (91.7%) and vomiting (71.7%) were the most frequently reported, followed by fever (53.3%). These values are within or higher than those observed in studies conducted in South Korea, China, Japan, and Thailand, where diarrhea was reported in 72% to 100% of cases, vomiting in 20% to 62%, and fever in 25% of children with AGE [43].

Although female children represented the group most affected by the virus (51.7%), no statistically significant association was found between infection and gender, suggesting that gender is not a determining factor in the establishment of HAstV infection. This finding is consistent with a study conducted in Shanghai that analyzed 6,051 samples and found no significant difference between sexes [44].

The highest detection rate in the present study was recorded in the state of Roraima (35.7%, 5/14), a considerably higher frequency compared to that observed in a previous study conducted in the same state between 2016 and 2017, which reported a rate of 2.4% in children under five years of age with AGE [40]. Tocantins had the lowest positivity rate (7.7%, 8/104), similar to the 6.9% positivity rate reported in a study conducted between 2010 and 2016 involving children of the same age group with AGE [21].

No HAstV-positive cases were detected in the state of Rondônia in the present study; however, the sample size from this state was very small (n = 6). Nevertheless, a previous study conducted between 2010 and 2012 involving the collection of 591 samples in Rondônia reported the presence of the virus at a rate of 0.8% [41], suggesting a lower circulation of HAstV in this state compared to others in the region.

The years with the lowest detection rates were 2020 and 2022, possibly due to the onset of the Coronavirus disease-19 (COVID-19) pandemic. During this period, the World Health Organization (WHO) declared the pandemic status of severe acute respiratory syndrome coronavirus 2 (SARS-CoV-2) infections, leading to a redirection of clinical efforts toward identifying COVID-19 cases. This shift likely resulted in underreporting of infections caused by other viral pathogens, including those responsible for diarrheal diseases [45,46].

In contrast, the highest positivity rate was observed in 2021 (23.1%). A similar trend was reported by Hoque et al. [47] in Japan’s Kansai region, where an analysis of enteric viruses in sewage samples collected between 2018 and 2022 showed continued circulation of such viruses. These findings support the hypothesis that, despite the reduction in reported clinical cases during the pandemic, infections with gastroenteric viruses like HAstV persisted, particularly in environmental reservoirs such as wastewater.

A study conducted in Japan by Okitsu et al. [48], using samples collected between 2014 and 2021 from children up to 15 years of age, reported findings similar to those of the present study. Notably, the sample size for the years 2020 and 2021 was significantly reduced, further supporting the hypothesis that the SARS-CoV-2 pandemic disrupted global health surveillance systems, including monitoring for HAstV. Similarly, in the presente study, the low number of HAstV detections observed in 2020 and 2022 may reflect the broader impact of the COVID-19 pandemic on healthcare access, sample submission, and surveillance activities, rather than a confirmed reduction in viral circulation.

When analyzed on a monthly basis, infection peaks were observed in May 2016, March 2017, and July 2018, but these patterns did not repeat in subsequent years, suggesting that HAstV does not exhibit a well-defined seasonality. However, the highest detection rates (11.6%) occurred during the so-called Amazonian winter, which spans from December to May and is characterized by increased rainfall. Similar results were reported in a study conducted in Brazil’s Northeast, Southeast, and South regions between 2005 and 2011, which also found higher HAstV occurrence during the rainy season [14].

In contrast, a study conducted in the state of Acre in 2012 [15] reported higher positivity during the warmer months. However, it is important to note that this study had a limited observation period (January to December 2012), whereas the present study spans a full decade, providing a more comprehensive understanding of the potential seasonality of HAstV – even though no statistically significant association was observed for this variable.

Over the 10-year study period, three classical HAstV genotypes (HAstV-1, HAstV-3, and HAstV-4) were identified circulating in the Northern region of Brazil, indicating low genotype diversity compared to findings from a study conducted in the Central-West region, where all eight classical serotypes were detected [49].

HAstV-1 is the predominant genotype reported in various regions around the world, although the second most common genotype may vary depending on the geographic area analyzed [7]. In Brazil, since the first reports of the virus in the northern region, HAstV-1 has remained the most frequently detected genotype. Similar detection rates have been reported for HAstV-3 (12.1%) in the state of Pará [39] and HAstV-4 (18.8%) in Tocantins [21].

Among the nine samples identified as HAstV-1, two exhibited 100% nucleotide similarity with strains previously registered in Brazil, including one from sewage samples collected in Rio de Janeiro in 2010 (JN799270.1) [50], and other one from children with acute gastroenteritis (AGE) in Tocantins in 2015 (MT832897.1, MW714406.1) [21]. The remaining seven HAstV-1 samples also showed high genetic identity, ranging from 99.24% to 99.80%, with strains registered in China in 2018 (MK579999.1), the United States in 2013 (MN433703.1), Thailand in 2017 (MG970101.1) – all from children with AGE symptoms – and Russia in 2017 (MG932590.1), from a 28-year-old adult.

Considering that the target regions of the primers used occupy distinct positions in the genome (ORF2 for Mon269/Mon270 and ORF1b for SF0073/SF0076), it is suggested that the divergence observed in this study during the characterization of the sample (AM/113/BR/03–2017) may be the result of either co-detection or a recombination event between HAstV-4 and HAstV-1 strains, likely occurring in the overlapping region between ORF1b and ORF2. However, despite the efforts undertaken, complete sequencing of this particular sample was not successful. This limitation is likely associated with the low viral load present in the fecal sample, which may have compromised the efficiency of the sequencing approach.

Since the first detection of the non-classical HAstV MLB-3 in samples collected in 2004 in India [51], this genotype has been reported in Gambia and Kenya in children with and without AGE symptoms [52], in Spain [53], in Japan in children with AGE [48], in Nigeria in children with acute flaccid paralysis [54], as well as in environmental samples, including sewage in Kenya [55], China [56], and Japan [57], and in river and groundwater samples in Nepal [58]. Until now, there has been no evidence of this genotype circulating in the Americas, making this the first report of HAstV MLB-3 on the continent and only the second full-genome deposit of this genotype in a public gene bank.

MLB-3 is considered a rare genotype compared to MLB-1 and −2 strains, with relatively few cases reported worldwide and low positivity rates when compared to classical genotypes. A positivity rate similar to that found in the present study (0.2%) has been reported in India (0.6%) [51], while higher rates have been observed in Gambia (3.1%) [52].

The pathogenesis of MLB-3 remains unclear, as it has been detected in both children with AGE [51] and asymptomatic individuals [52]. The MLB-3–positive sample identified in this study was obtained from a two-year-old male child presenting symptoms of vomiting and diarrhea, but no fever. Information on vaccination status for other pathogens was unavailable. This clinical profile differs from that reported in a case in Japan, where a child infected with MLB-3 exhibited all three symptoms: vomiting, diarrhea, and fever [48].

The MLB-3–positive sample was collected in the state of Amazonas in February 2014, indicating that this genotype was present in Brazilian territory at that time. However, alternative explanations should be considered, including a sporadic introduction event, such as zoonotic transmission or an imported case, without evidence of sustained circulation. Nevertheless, this finding underscores the importance of continuous molecular epidemiological surveillance of this virus, particularly given that it is an RNA virus with high mutation rates, which may confer broader clinical and epidemiological relevance in the future depending on its evolutionary adaptation to the host, as observed for other viral agents.

Future studies should expand whole-genome sequencing efforts for both classical and non-classical HAstV strains circulating in Brazil, particularly to investigate potential recombination events and to better define the evolutionary relationships of rare genotypes such as MLB-3. In addition, broader virome-based approaches, including metagenomic analyses, may help clarify possible coinfections and the ecological context of HAstV in pediatric gastroenteritis. Whenever feasible, prospective studies integrating clinical severity, viral load, and genomic characterization may further improve understanding of the role of HAstV in childhood AGE in the Amazon region.

No non-classical HAstVs from the VA clade were detected in the present study, consistent with findings from other studies conducted in children under six years of age in the Northern Region between 2016 and 2017 [40]; in the Northeast, Southeast, and South regions between 2005 and 2011 [14]; and in the Central-West between 2014 and 2015 [59]. In those studies, only MLB-1 and −2 genotypes were identified, suggesting that HAstV VA clade strains are not yet circulating in Brazilian territory.

## Conclusion

- This study provides new data on the epidemiological profile and genetic diversity of both classical and non-classical HAstVs in children from Northern Brazil between 2013 and 2022. The positivity rate for classical HAstVs detected via RT-qPCR was relatively higher than the rates reported, though lower than rates reported in other regions using conventional RT-PCR. Likewise, the frequency of non-classical HAstVs was lower compared to findings in other parts of the world.
- Infection was more prevalent among children aged 5–10 years, with no significant differences between sexes. While HAstV infections did not exhibit a consistent annual distribution pattern, higher detection rates were observed during the Amazonian winter season, with the state of Roraima reporting the highest number of cases.
- Genotypic analysis revealed the circulation of HAstV-1, HAstV-3, and HAstV-4. Importantly, this study reports, for the first time, the detection of the non-classical HAstV MLB-3 genotype in the Americas, identified in the fecal sample of a two-year-old child from the state of Amazonas.

## Supporting information

S1 Fig Phylogenetic tree constructed from 42 partial ORF2 sequences (336 bp) of classical HAstV strains detected in fecal samples from children with gastroenteritis in Northern Brazil, from 2013 to 2022. The tree was constructed using the Maximum Likelihood method with nonparametric bootstrap testing (1,000 replicates), and the evolutionary history was inferred by Bayesian inference (MrBayes) using the GTR substitution model in Geneious software. Strains analyzed in this study are highlighted in red and identified by Brazilian federative unit, sample number, country, and collection date.
(TIF)

S2 Fig Phylogenetic tree constructed from the complete genome sequence (6,117 bp) of the non-classical HAstV-MLB-3 genotype detected in February 2014, in a fecal sample from a child with gastroenteritis in the state of Amazonas, Northern Brazil. The genome was obtained using a target enrichment approach based on hybrid capture, followed by sequencing on the Illumina MiSeq™ platform. Raw reads were processed and trimmed using FASTP, assembled de novo with MEGAHIT, and aligned using MAFFT, with genome editing performed in Geneious software. Strains analyzed in this study are highlighted in bold.
(PNG)
