# Supplementary figures and images for: Astrovirus in the Brazilian Amazon: First detection of non-classical astroviruses (MLB-3) in the Americas
Source: PLoS One. 2026 Jul 1;21(7):e0352094. doi: 10.1371/journal.pone.0352094 (PMC13322526; doi:10.1371/journal.pone.0352094)

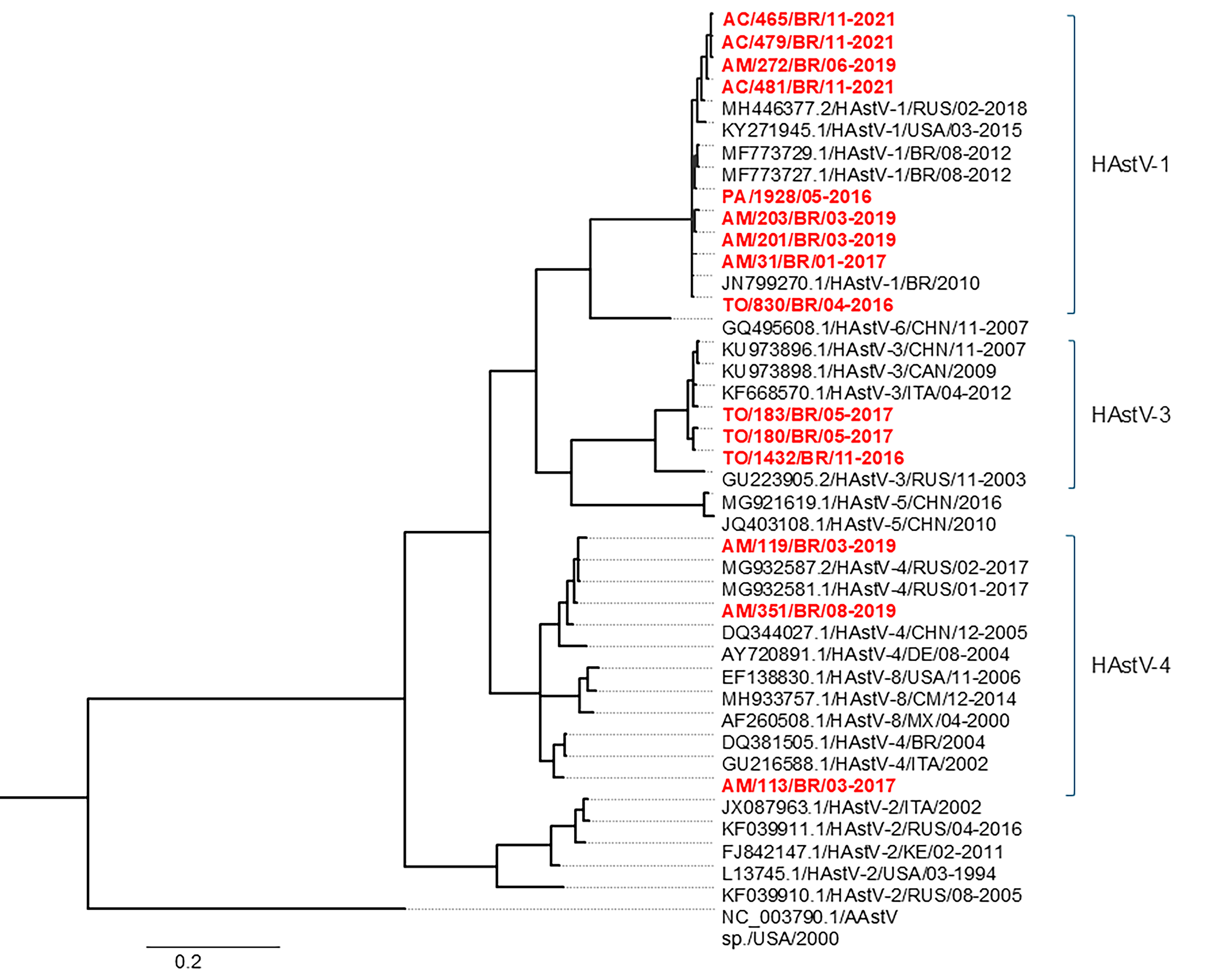

Supplement: S1 Fig — The tree was constructed using the Maximum Likelihood method with nonparametric bootstrap testing (1,000 replicates), and the evolutionary history was inferred by Bayesian inference (MrBayes) using the GTR substitution model in Geneious software. Strains analyzed in this study are highlighted in red and identified by Brazilian federative unit, sample number, country, and collection date. (TIF) [file pone.0352094.s001.tif]

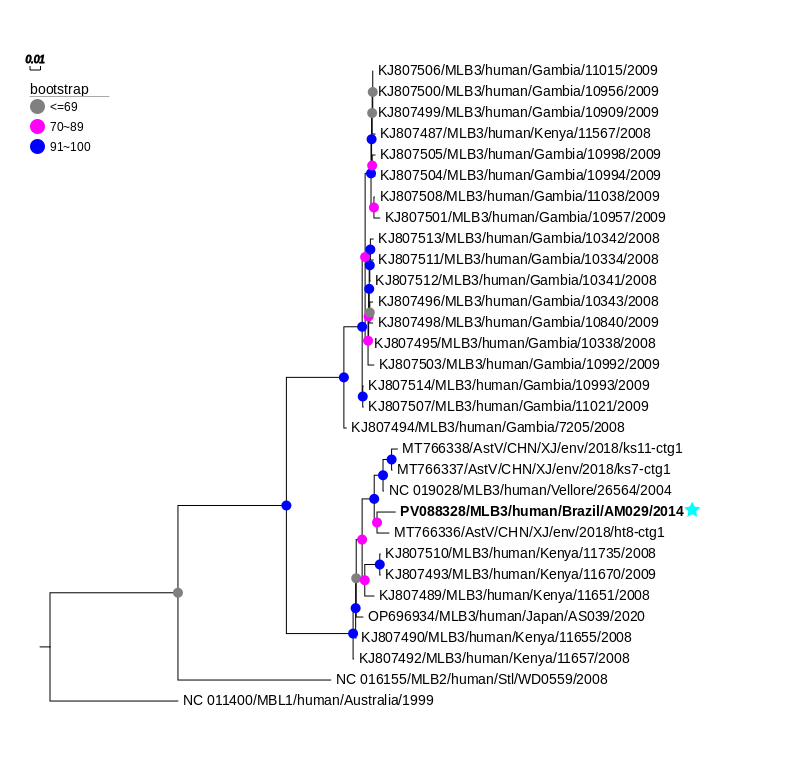

Supplement: S2 Fig — The genome was obtained using a target enrichment approach based on hybrid capture, followed by sequencing on the Illumina MiSeq™ platform. Raw reads were processed and trimmed using FASTP, assembled de novo with MEGAHIT, and aligned using MAFFT, with genome editing performed in Geneious software. Strains analyzed in this study are highlighted in bold. (PNG) [file pone.0352094.s002.png]
